# Analysis of macular thickness and peripapillary retinal nerve fiber layer thickness in various ABO and Rh blood groups

**DOI:** 10.1186/s12886-024-03577-5

**Published:** 2024-07-24

**Authors:** Effat Shanbezadeh, Behnoosh Vasaghi-Gharamaleki, Payam Nabovati, Leili Koochakzadeh, Mehdi Khabazkhoob

**Affiliations:** 1 Noor Ophthalmology Research Center, Noor Eye Hospital, Tehran, Iran; 2 Rehabilitation Research Center, Department of Basic Sciences in Rehabilitation, School of Rehabilitation Sciences, Iran University of Medical Sciences, Tehran, Iran; 3 Rehabilitation Research Center, Department of Optometry, School of Rehabilitation Sciences, Iran University of Medical Sciences, Tehran, Iran; 4 Department of Pediatrics, School of Medicine, Childrens Medical Center, Tehran University of Medical Sciences, Tehran, Iran; 5 Department of Medical Surgical Nursing, School of Nursing and Midwifery, Shahid Beheshti University of Medical Sciences, Tehran, Iran

**Keywords:** Blood group, Retinal structural indices, Macular thickness, Retinal nerve fiber layer thickness

## Abstract

**Purpose:**

To determine the association between ABO and Rh blood groups with retinal structural indices including macular thickness and peripapillary retinal nerve fiber layer (RNFL) thickness.

**Methods:**

This cross-sectional study was conducted using convenience sampling in a tertiary referral eye hospital in Tehran, Iran. Study participants were referred to the hospital laboratory to test their blood group. Ocular examinations were performed including measurement of visual acuity, auto-refraction, subjective refraction, and slit-lamp biomicroscopy. Retinal imaging was carried out using Spectral-domain OCT under dilated papillary conditions.

**Results:**

Three hundred and twenty-eight individuals were recruited in this study. Of these, 219 (60.7%) were female and the mean age of the participants was 63.29 ± 5.57 years (range: 56 to 83 years). According to the multiple linear regression model, the global peripapillary RNFL thickness [coefficient: -3.05 (95% CI: -5.30 to -0.74); *P* = 0.010] and peripapillary RNFL thickness in the superior [coefficient: -4.65 (95% CI: -8.40 to -0.89), *P* < 0.001] and inferior [coefficient: -4.00 (95% CI: -7.81 to -0.19); *P* = 0.040] quadrants were significantly thinner in individuals with blood type B compared to those with other ABO blood groups. The average [coefficient: 12.69 (95% CI: 4.12–21.64); *P* = 0.004) and central [coefficient: 16.21 (95%: 6.44–25.97); *P* = 0.001) macular thicknesses were significantly thicker in AB group compared to other blood groups. The average macular thickness was significantly thinner in Rh + compared to the Rh- group [coefficient: -8.33 (95% CI: -15.4 to -1.25); *P* = 0.021].

**Conclusion:**

Retinal structural indices may be related to blood groups implying a genetic linkage. Considering the lack of consistency among various studies, larger trials are needed to explore the effect of ABO and Rh grouping on peripapillary RNFL and macular thicknesses.

## Introduction

The ABO blood group system consisting of four antigens (A, B, AB, and O) was discovered in 1900 by the Austrian scientist Karl Landsteiner [1, 2]. These antigens are known as oligosaccharide antigens and are widely expressed on the membranes of red blood cells and tissue cells, as well as in saliva and body fluids [2]. In 1940, Landsteiner and Wiener discovered a new antigen (Rh) in human red blood cells [3]. The Rh is one of the critical blood antigens, clinically next to ABO antigens [4]. Blood type is a genetically determined factor suggested as a risk factor for many diseases [5]. The results of various studies have confirmed the relationship between blood groups and most vascular diseases [6–9]. 

The expression of genes in the cornea is mostly related to the pathways associated with protein synthesis, cellular turnover, and cellular defenses, while the genes expressed in the retina more than in the cornea are involved in the pathways related to blood vessels and photo-transduction [10]. Gene expression occurs more in the cornea and retina than in other ocular parts [11]. Since gene expression in the retina is equal to [10] or more [11, 12] than that of the cornea, and the cornea is an avascular structure, [12] this could indicate the importance of genetics role and blood groups in the retina compared to other ocular tissues. Some conditions such as retinal detachment (RD), color vision deficiency, age-related macular degeneration (AMD), diabetic retinopathy, and retinitis pigmentosa (RP) are important retina-related diseases or disorders that previous studies have shown their genetic linkages [13–19]. 

The retinal structural parameters including peripapillary retinal nerve fiber layer (RNFL) thickness and macular thickness have also shown a relationship with genetic factors [20, 21]. The genetic pool determines the blood type; [22] on the other hand, retinal diseases and characteristics are related to genetics. Therefore, a theoretical relationship can be proposed between blood type with retinal diseases and retinal structural indices. There are limited and inconsistent reports on the relationship between ABO blood groups with retinal diseases or indices. Previous studies in this field were mostly limited to inter-group comparisons and failed to effectively control confounding factors. The present study aimed to determine the relationship between ABO blood groups and the Rh system with retinal structural indices including macular and peripapillary RNFL thicknesses.

## Methods

### Study design and sampling

The present cross-sectional study was conducted in a tertiary referral eye hospital in Tehran, Iran. This referral eye hospital was chosen for the study as the equipment needed for ocular examination and imaging as well as laboratory were all available in this center. Study samples were selected using the convenience sampling method from those referred to the hospital for eye examination between December 2022 and May 2023.

The sample size was calculated based on a study by Teberik et al., [23] based on the difference in the average macular thickness between B and O blood groups which showed the largest difference. The sample size was estimated at 35 individuals in each blood group according to the averages reported in the above study (341.1 ± 13.6 and 331.0 ± 16.2 microns), the alpha error = 0.50, and the study power = 80%. Considering a 20% sample drop, the sample size was increased to 42 people in each of the eight blood groups (A^+^, A^−^, B^+^, B^−^, AB^+^, AB^−^, O^+^, and O^−)^, and the final sample size was considered equal to 336 people.

This study followed the tenets of the Declaration of Helsinki. The study protocol was approved by the Ethics Committee of the Iran University of Medical Sciences (ethics code: IR.IUMS.REC.1401.669). The study goals and steps were fully explained to all invitees before participation. Written informed consent was obtained from all participants. First, the blood type was inquired based on the participant’s report or observing driving license card information. The participants were then referred to the hospital laboratory for blood testing to confirm their blood group and measure hemoglobin A1c (HbA1c) level. The sampling continued until the required sample size was completed across different blood groups.

#### Examinations

Demographic and case history information was collected through an interview. The body mass index (BMI) was calculated after measurements of height and weight using the following formula: weight (kg)/height (m^2^). Systolic and diastolic blood pressures (SBP and DBP) were measured using sphygmomanometer (OMRON, HEM-2228-E, Kyoto, Japan) twice, 10 min apart, and the average was recorded.

Preliminary optometric examinations included measurement of uncorrected distance visual acuity (UCVA) using the Nidek CP-770 chart projector (Nidek, Gamagori, Japan) at 6 m (m), auto-refraction using an auto-refractometer (ARK-1, Nidek Co., Aichi, Japan), and subjective refraction to determine optimal distance optical correction and the best-corrected distance visual acuity (BCVA). In the next step, study participants underwent ocular biometry using IOL Master 500 (Carl Zeiss Meditec AG, Jena, Germany); three high-quality axial length (AL) measurements with a signal-to-noise ratio (SNR) above 2.0 were obtained and the average of three measurements was recorded. Then, anterior and posterior segment ocular examination was performed by an ophthalmologist using a slit-lamp biomicroscope (B900, Haag-Streit AG, Bern, Switzerland) and a + 90 D lens (Volk Optical Inc, Mentor, OH, USA). Intraocular pressure (IOP) was measured using Goldmann Applanation tonometry (GAT; Haag-Streit AG, Köniz, Switzerland).

Retinal imaging was carried out using Spectral-domain OCT (Spectralis, Heidelberg Engineering, Heidelberg, Germany) by a single experienced operator following cycloplegia with two drops of Tropicamide 1% (Mydrax; Sina Darou, Tehran, Iran). The instrument’s automatic real-time eye tracker was used to eliminate motion artifacts during scanning. Two sets of scans were obtained (a 20 × 20° macular volume scan and an RNFL optic disc scan) and the outputs presenting peripapillary RNFL thickness, macular thickness, and macular volume were automatically generated. Macular thickness was classified according to the Early Treatment Diabetic Retinopathy Study (ETDRS) grid; central foveal circle (diameter = 1 mm), parafoveal or inner circle (diameter = 3 mm), and perifoveal or outer circle (diameter = 6 mm). The parafoveal and perifoveal regions were further divided into superior, inferior, temporal, and nasal subfields. The peripapillary RNFL thickness map comprised a global average and average thickness of the four quadrants; superior, inferior, nasal, and temporal. Only scans with a Q score > 15 dB were considered valid.

#### Inclusion and exclusion criteria

Inclusion criteria were age ≥ 55 years and a BCVA of 20/20 in both eyes. Exclusion criteria were a history of any genetic or systemic diseases affecting the retina and optic nerve (e.g. diabetes, hypertension, Marfan syndrome), HbA1c ≥ 6.5%, SBP ≥ 140 mm Hg and/or DBP ≥ 90 mm Hg, using drugs affecting the retina and optic nerve such as hydroxychloroquine, history of intraocular surgery, degenerative myopia (spherical equivalent refraction<-6.00 diopter), history of ocular trauma, signs of retinal or optic nerve diseases in fundus examination (e.g., retinal tears, hemorrhages, microaneurysms, macular hole, exudates, arterial and venous abnormalities, cup/disk ratio > 0.50, neuroretinal rim loss), intraocular pressure above 21 mmHg, significant media opacity (corneal opacity and cataracts more than grade 1 according to LOCS III classification system).

### Statistical analysis

Statistical analysis was performed using Statistical Package for the Social Sciences (SPSS) version 26 (IBM Inc., Chicago, IL, USA). The mean and standard deviation (SD) of macular thickness, macular volume, and peripapillary RNFL thickness was reported in different ABO and Rh blood groups. One-way analysis of variance and independent samples t-test were used to compare these thickness parameters between various ABO and Rh blood groups. The relationship between thickness indices with ABO and Rh groups was investigated using multiple linear regression models by controlling the effect of confounding variables. Age, sex, AL, BMI and IOP were considered potential confounders based on their associations with macular or peripapillary RNFL thicknesses in previous studies. A p-value less than 0.05 was considered statistically significant.

### Ethical consideration

This project was supported by Iranian University of Medical Sciences (IUMS) and followed the tenets of the Declaration of Helsinki. The protocol of the study was approved by the Ethics Committee of Iran University of Medical Sciences (ethical code: IR.IUMS.REC.1401.669). Informed consent was obtained from all participants.

## Results

Three hundred and twenty-eight individuals were recruited in this study. Due to the rarity of the blood group AB^−^, only 34 people with this blood type were identified during the study period. Therefore, of the 42 individuals required in each blood group based on the sample size calculation, 34 people could be provided for blood group AB^−^. The other seven blood groups met the desired sample size. The mean age of the participants was 63.29 ± 5.57 years (range: 56 to 83 years), and 60.7% (219) were female.

There was a high correlation between the right and left eyes in the average macular thickness (Pearson’s correlation coefficient = 0.863) and the average peripapillary RNFL thickness (Pearson’s correlation coefficient = 0.880); therefore, only the results of the right eye were analyzed in this report.

Table 1 shows the distribution of some ocular and demographic variables by ABO blood and Rh groups.

**Table 1:** The distribution of some ocular and demographic variables by ABO blood and rh groups

	Blood groups				Rh		
	A	B	AB	O	Negative	Positive	All
	Mean ± SD	Mean ± SD	Mean ± SD	Mean ± SD	Mean ± SD	Mean ± SD	Mean ± SD
Age (years)	63.23 ± 5.66	63.26 ± 6.02	63.51 ± 5.4	63.19 ± 5.2	63.83 ± 5.41	62.85 ± 5.67	63.29 ± 5.57
Axial length (mm)	23.25 ± 1.12	23.09 ± 1.22	23.47 ± 1.56	23.18 ± 0.93	23.39 ± 1.33	23.11 ± 1.11	23.24 ± 1.22
Intraocular pressure (mmHg)	15.98 ± 2.47	15.23 ± 2.17	15.18 ± 2.62	15.78 ± 2.38	16.56 ± 2.6	14.79 ± 1.97	15.55 ± 2.42
Body mass index (kg/m^2^)	28.68 ± 3.61	29.44 ± 4.39	27.62 ± 3.77	29.03 ± 4.5	28.45 ± 3.81	28.97 ± 4.35	28.73 ± 4.12
Education (years)	8.14 ± 5.04	8.36 ± 5.25	7.46 ± 4.97	9.45 ± 7.24	7.94 ± 5.19	8.73 ± 6.11	8.37 ± 5.72
Gender (% of female)	64.60%	66.00%	53.20%	57.30%	59.30%	61.80%	60.70%

Table 2 shows the mean ± SD of the average, central, perifoveal (inner), and parafoveal (outer) thicknesses as well as macular volume in different ABO and Rh blood groups. One-way ANOVA showed statistically significant differences in the average and central macular thicknesses between ABO blood groups (p-values: 0.022 and 0.006, respectively); the highest and lowest average and central thicknesses were observed in participants with blood groups AB and A, respectively. The average and central macular thickness were significantly higher in Rh^+^ compared to the Rh^−^ group (p-values: 0.041 and 0.022, respectively). There were no statistically significant differences in the perifoveal and parafoveal thicknesses as well as the macular volume between ABO and Rh groups (all p-values > 0.05).

**Table 2:** Mean ± standard deviation of the average, central, perifoveal, and parafoveal macular thicknesses and macular volume in different ABO and rh blood groups

Macular thickness (µ)	ABO blood groups					Rh group		
	**A**	**B**	**AB**	**O**	p-value*	**+**	**-**	p-value**
	**mean ± SD**	**mean ± SD**	**mean ± SD**	**mean ± SD**		**mean ± SD**	**mean ± SD**	
Average	264.2 ± 32.7	269.1 ± 30.8	280.3 ± 48.8	268 ± 27.1	0.022	274.3 ± 40.2	266.4 ± 31	0.041
Central	217.6 ± 32.8	223.4 ± 33	237.4 ± 59	220.5 ± 27.8	0.006	229.6 ± 47.1	219.7 ± 31.8	0.022
Inner superior	334.5 ± 31.4	334.1 ± 19.1	332.9 ± 22.1	337.3 ± 37.8	0.787	334.2 ± 27.2	335.2 ± 29.9	0.758
Inner temporal	321.4 ± 22.3	323.5 ± 28.2	325.4 ± 21.1	322 ± 19.4	0.667	323.3 ± 20.3	322.7 ± 25.1	0.795
Inner nasal	333.9 ± 21.9	336.2 ± 19.3	337.3 ± 26.1	335.1 ± 16.8	0.739	335.7 ± 21.2	335.4 ± 21.1	0.92
Inner inferior	329.1 ± 20.9	330.1 ± 24.8	333.5 ± 27.2	332.1 ± 17.3	0.572	330.5 ± 20.3	331.6 ± 24.5	0.642
Outer superior	289.6 ± 29.5	285.2 ± 17.5	285.4 ± 18.3	287.2 ± 16.3	0.471	287.4 ± 25.8	286.6 ± 17	0.73
Outer temporal	277.5 ± 19.9	275.5 ± 25.4	277 ± 16.9	276 ± 16.3	0.901	276.7 ± 18.8	276.3 ± 21	0.831
Outer nasal	305 ± 20.7	302.8 ± 18.0	300.6 ± 22.1	302.4 ± 16.7	0.502	302.2 ± 19.9	303.3 ± 19.1	0.617
Outer inferior	277.5 ± 20.5	277.8 ± 16.9	279.1 ± 22	278.8 ± 21	0.945	276.6 ± 20.3	279.6 ± 19.8	0.171
Macular volume (mm^3^)	8.33 ± 0.63	8.34 ± 0.52	8.36 ± 0.56	8.36 ± 0.43	0.965	8.33 ± 0.58	8.36 ± 0.51	0.537

* The p-value was calculated by one-way analysis of variance

** The p-value was calculated by independent samples t-test

The mean ± SD of the global and sectoral peripapillary RNFL thicknesses in different ABO and Rh blood groups are presented in Table 3. According to Table 3, there were statistically significant differences in the global and all sectoral peripapillary RNFL thicknesses between ABO blood groups (all p-values < 0.005); the lowest mean global and sectoral peripapillary RNFL thicknesses were related to participants with blood type B. Participants with blood type Rh^−^ had a significantly higher mean peripapillary RNFL thickness in the inferior quadrant compared to those with blood type Rh^+^ (p-value = 0.002).

**Table 3:** Mean ± standard deviation of the global and sectoral RNFL thicknesses in different ABO and rh blood groups

RNFL thickness (µ)	ABO blood group					Rh group		
	A	B	AB	O		+	-	
	mean ± SD	mean ± SD	mean ± SD	mean ± SD	*p*-value*	mean ± SD	mean ± SD	*p*-value**
Global	101.25 ± 9.08	94.08 ± 8.37	96.54 ± 11.95	100.24 ± 8.66	< 0.001	97.26 ± 9.29	98.79 ± 10.38	0.144
Temporal	71.71 ± 10.62	67 ± 8.19	71.75 ± 16.33	69.12 ± 11.24	0.016	69.5 ± 12.33	70.14 ± 11.49	0.610
Superior	123.92 ± 15.86	113.06 ± 14.02	115.68 ± 17.4	122.36 ± 14.45	< 0.001	119.03 ± 15.9	118.8 ± 16.18	0.896
Nasal	78.78 ± 12.27	74.31 ± 11.53	74.62 ± 14.05	79.43 ± 14.66	0.011	76.58 ± 12.92	77.09 ± 13.58	0.717
Inferior	130.64 ± 16.3	121.76 ± 17.12	124.89 ± 16.41	130.14 ± 15.51	< 0.001	123.94 ± 15.42	129.39 ± 17.36	0.002

RNFL: Retinal Nerve Fiber Layer

*: The p-value was calculated by one-way analysis of variance

**: The p-value was calculated by independent samples t-test

Table 4 shows the results of multiple regression models for the associations of peripapillary RNFL thickness with ABO blood groups after controlling potential confounders (age, sex, AL, BMI, and IOP). As seen in Table 4, the global peripapillary RNFL thickness [coefficient: -3.05 (95% CI: -5.30 to -0.74); p-value = 0.010] and peripapillary RNFL thickness in the superior [coefficient: -4.65 (95% CI: -8.40 to -0.89), p-value < 0.001] and inferior [coefficient: -4.00 (95% CI: -7.81 to -0.19); p-value = 0.040] quadrants were significantly thinner in individuals with blood type B compared to those with other ABO blood groups. Superior quadrant peripapillary RNFL thickness was significantly thinner in participants with blood type AB compared to those with other ABO blood groups [coefficient: -4.71(95% CI: -8.65 to -0.79); p-value = 0.019]. Global peripapillary RNFL thickness [coefficient: 2.8 (95% CI: 0.49 to 5.14); p-value = 0.018] and peripapillary RNFL thickness in the superior [coefficient: 4.32 (95% CI: 0.55 to 8.10); p-value = 0.025] and inferior [coefficient: 3.97 (95% CI: 0.38 to 7.56); p-value = 0.030] quadrants were significantly thicker in participants with blood type O compared to those with other ABO blood groups. None of the peripapillary RNFL thickness indices showed a statistically significant relationship with the Rh factor in the multiple regression model (all p-values > 0.05).

**Table 4:** Multiple linear regression models for the associations of RNFL thickness with ABO blood groups after controlling for age, sex, axial length, body mass index, and intraocular pressure

RNFL thickness (µ)	Blood group				
	A	B	AB	O	
	Coefficient (95% CI); *p*-value	Coefficient (95% CI); *p*-value	Coefficient (95% CI); *p*-value	Coefficient (95% CI); *p*-value	
Global	NS	-3.05 (-5.30 to -0.74); 0.010	NS	2.8 (0.49 to 5.14); 0.018	
Temporal	NS	NS	NS	NS	
Superior	NS	-4.65 (-8.40 to -0.89); <0.001	-4.71 (-8.65 to -0.79); 0.019	4.32 (0.55 to 8.10); 0.025	
Nasal	NS	NS	NS	NS	
Inferior	NS	-4.00 (-7.81 to -0.19); 0.040	NS	3.97 (0.38 to 7.56); 0.030	

RNFL: Retinal Nerve Fiber Layer

CI: confidence interval

NS: not significant

According to the multiple regression model after controlling the effect of potential confounders (age, sex, AL, BMI, and IOP), the average [coefficient: 12.69 (95% CI: 4.12–21.64); p-value = 0.004) and central [coefficient: 16.21 (95%: 6.44–25.97); p-value = 0.001) macular thicknesses were significantly thicker in AB group compared to other blood groups. Moreover, the average macular thickness was significantly thinner in Rh^+^ compared to the Rh^−^ group [coefficient: -8.33 (95% CI: -15.4 to -1.25); p-value = 0.021].

## Discussion

The present study investigated the association between ABO and Rh blood groups with retinal structural indices (macular and peripapillary RNFL thicknesses) measured by SD-OCT in an adult population. The results showed a significantly higher average and central macular thickness in participants with blood type AB compared to those with other ABO blood groups. Moreover, the average macular thickness was significantly thinner in Rh^+^ compared to the Rh^−^ group. Teberik and Eski [23] found a significant difference in the temporal retinal thickness at a distance of 1000 microns from the fovea between ABO groups so that blood groups B and AB had the highest (341.1 µ) and the lowest (322.5 µ) mean thicknesses, respectively. Various factors may be involved in this discrepancy, including different age distributions (27.7 vs. 63.3 years), different inclusion and exclusion criteria, heterogeneity of the sample size across blood groups as well as the lack of control over confounding factors in the Teberik and Eski’s study. Since Teberik and Eski’s study [23] was limited to a simple retinal thickness comparison between blood groups, it cannot show an accurate picture of the association between blood groups and macular thickness. Although it is difficult to explain a thicker fovea in individuals with blood type AB, it could be due to a genetic link. Epidemiological studies have shown racial differences in macular thickness. Accordingly, macular thickness has been reported to be higher in Caucasians, followed by Asians and Africans, respectively. Macular thickness has been shown to be a highly heritable trait [24, 25]. In a study by Chamberlain et al., [26] heritability estimates were 85%, 81%, and 81% for foveal thickness, inner macular thickness, and outer macular thickness, respectively, indicating that macular thickness is predominantly influenced by genetics rather than environmental factors. A genome-wide association analysis identified 139 loci associated with macular thickness in the UK Biobank cohort comprising 68,423 participants. The blood group is also genetically determined; the ABO gene is located on chromosome 9 and has three alleles: A, B, and O. Based on the results of the above genome-wide association study, one of the top-ranking gene loci associated with an increased macular thickness (Beta: 0.72) was MIR31HG – MTAP on chromosome 9 [27]. Apart from the genetic link, differences in some biochemical characteristics between different blood groups may also contribute to this finding. In the present study, blood type B was significantly related to a decreased global peripapillary RNFL thickness and sectoral peripapillary RNFL thicknesses in the superior and inferior quadrants. Blood type AB was associated with a decreased peripapillary RNFL thickness in the superior quadrant. Individuals with blood type O showed thicker global peripapillary RNFL thickness as well as superior and inferior peripapillary RNFL thicknesses compared to other ABO blood groups. To our best knowledge, this is the first study to examine the relationship between blood groups and peripapillary RNFL thickness in a healthy population. Previous studies mainly focused on the association between ABO blood groups and glaucoma [28–31]. Although the findings are inconsistent around this subject, most of the previous studies have pointed to the relationship between blood type B and increased risk of glaucoma or higher prevalence of glaucoma in people with blood type B, which is somehow in line with the present study. Leske et al. [29] did not report any relationship between primary open-angle glaucoma (POAG) and the ABO blood groups. According to a study by Garg and Pahwa, [31] the frequencies of blood groups A and B were significantly higher, while the frequencies of blood groups AB and O were lower in patients with POAG and primary angle closure glaucoma (PACG). Zaree et al. [28] reported that blood group B was more prevalent in primary congenital glaucoma compared to controls. Imran Khan et al. [30] found a significant relationship between blood type B and different types of glaucoma. By combining the findings of the present study and previous reports, it can be concluded that a thinner peripapillary RNFL in people with blood type B may make them more susceptible to glaucomatous damage. Therefore, the association between glaucoma and blood type B may be routed in the optic nerve structure. This association may be due to a genetic link as peripapillary RNFL thickness has been shown to have a relatively high heritability. Hougaard et al. [32] reported heritability score for peripapillary RNFL thickness to be as high as 0.82 in a twin study. Van Koolwijk et al. [21] found the peripapillary RNFL thickness heritability score to be 0.48 in a family study of 2620 participants. A possible explanation is that the peripapillary RNFL thickness-related loci have been mapped to chromosome 9, which also contains the loci for the ABO blood group antigen. It may also be possible that glycosyltransferases, which are expressed in individuals with the B antigen, have some association with peripapillary RNFL thickness.

The association of ABO blood groups with macular and peripapillary RNFL thicknesses has important clinical implications. First, since macular thickening is an important diagnostic sign of macular disease; it is recommended that blood type be considered in the clinical interpretation of macular thickness, especially in people with blood type AB, to avoid misdiagnosis. Second, the peripapillary RNFL thickness measurements are color-coded and reported as within normal limits, borderline, or outside normal limits in the OCT printout based on a normal database. Considering inherent peripapillary RNFL thinning, one should be cautious in interpreting abnormal reports in individuals with blood type B to avoid false positive diagnoses of optic neuropathy. Third, people with blood type B may be more susceptible to significant peripapillary RNFL loss and advanced glaucomatous damage due to an inherent thin peripapillary RNFL, so it is logical to be more careful in the clinical management of these patients in terms of follow-up periods and IOP control. The two later points should be emphasized since the significant relationship between blood group and peripapillary RNFL thickness was observed in the vertical quadrants, the areas that are specifically affected in glaucoma.

The present study has limitations. Although we tried to effectively control the confounding factors, there may still be other uncontrolled confounders. The hospital-based nature of the study reduces its generalizability. The sampling method itself may have added bias. As the study was performed in an elderly population, the conclusions may not be applicable to other age groups. We failed to investigate the effect of two B alleles (BB) versus one B allele (BO) on the peripapillary RNFL thickness.

## Conclusion

The authors found a direct correlation of the average and central macular thickness with blood group AB, and an inverse correlation of the global and vertical peripapillary RNFL thicknesses with blood group B. Since there is a lack of agreement among various studies, the findings regarding the relationship between blood groups and retinal structural indices may be incidental. Larger trials are needed to explore the effect of ABO and Rh grouping on peripapillary RNFL and macular thicknesses.

## Data Availability

No datasets were generated or analysed during the current study.
